# Loneliness among people living with dementia: results from the English Longitudinal Study of Ageing

**DOI:** 10.1002/alz.088901

**Published:** 2025-01-09

**Authors:** Thamara Tapia‐Muñoz, Claudia Miranda‐Castillo

**Affiliations:** ^1^ Millennium Institute for Care Research, Santiago Chile; ^2^ University College London, London United Kingdom; ^3^ Millennium Nucleus on Sociomedicine, Santiago Chile; ^4^ Universidad Andrés Bello, Santiago, Santiago Chile; ^5^ Millenium Institute for Research in Depression and Personality, Santiago Chile

## Abstract

**Background:**

Literature often focuses on loneliness as a risk factor for dementia. However, loneliness among those living with dementia (PLWD) is yet to be further explored.

**Method:**

Secondary analysis of the English Longitudinal Study of Ageing. Across ELSA waves 1 to 9, we identified 181 participants who reported being diagnosed with dementia and randomly selected 775 (10%) non‐dementia participants (NDP) from wave 2 (2004‐2005). For PLWD, the baseline was the year they reported the diagnosis. For NDP, the baseline was information from waves 1 and 2. We obtained a two‐year lagged loneliness for a subsample (n = 70 PLWD and n = 569 NDP). We computed multiple linear regression models using bootstrap errors with 1000 iterations to describe the average levels of loneliness among people living with dementia in England.

**Result:**

Both groups did not differ in their socioeconomic characteristics except for age. PLWD were, on average, 76 years old (Sd = 9.74), and NDP was 66 years old (sd = 9.53). The average level of loneliness was 4.71 (sd = 1.74) for PLWD and 4.07 (sd = 1.47) for NDP. At baseline, the loneliness prevalence was 15.76% among PLWD and 7.55% for NDP. Among PLWD, the average 2‐year lagged loneliness was 4.72 (sd = 1.79), and 21% reported problematic loneliness, and among NDP, it was 4.18 (sd = 1.52) and 10%, respectively. Adjusted baseline levels of loneliness were statistically significantly higher among PLWD (Coef. = 0.694; 95% CI: 0.393‐0.994). For the 2‐year lagged loneliness, dementia and non‐dementia participants did not significantly differ in their levels of loneliness.

**Conclusion:**

Public policies should consider tackling loneliness among people living with dementia.

